# Alveolar Paratesticular Rhabdomyosarcoma in an Adult Patient With PAX3-FOXO1 Fusion and Unfavorable Evolution

**DOI:** 10.7759/cureus.71279

**Published:** 2024-10-11

**Authors:** Eduardo O Paese, Rubens Rodriguez, Alan A Azambuja

**Affiliations:** 1 College of Medicine, Pontifícia Universidade Católica Do Rio Grande Do Sul, Porto Alegre, BRA; 2 Pathology, Hospital Mãe De Deus, Porto Alegre, BRA; 3 Oncology, Pontifícia Universidade Católica Do Rio Grande Do Sul, Porto Alegre, BRA

**Keywords:** paratesticular sarcoma, pax3-foxo1, testis cancer, urooncology, uropathology

## Abstract

Rhabdomyosarcomas, malignant mesenchymal tumors of skeletal striated muscle tissue cells, are usually rare in adults. However, when they occur in this population, the prognosis is usually poor, especially if the condition is associated with molecular factors such as the PAX3-FOXO1 fusion. Here, We report a case of paratesticular alveolar rhabdomyosarcoma in an adult patient who initially complained of increased scrotal volume for two years and presented with a PAX3-FOXO1 fusion. This emphasizes the dire prognosis of the disease, reinforcing the need for thorough and directed diagnostic efforts.

## Introduction

Rhabdomyosarcomas are malignant mesenchymal tumors that originate from skeletal striated muscle cells, of which approximately 20% arise in the genitourinary tract [1,2]. Because it is rare in adults, considerably more common differential diagnoses, such as seminomas, may initially prevail, as clinical manifestations are similar, including the presence of a mass or swelling, generally painless [3]. Here, we report the case of an adult patient being investigated for rhabdomyosarcoma after initial suspicion of seminoma.

## Case presentation

**Figure 1:**
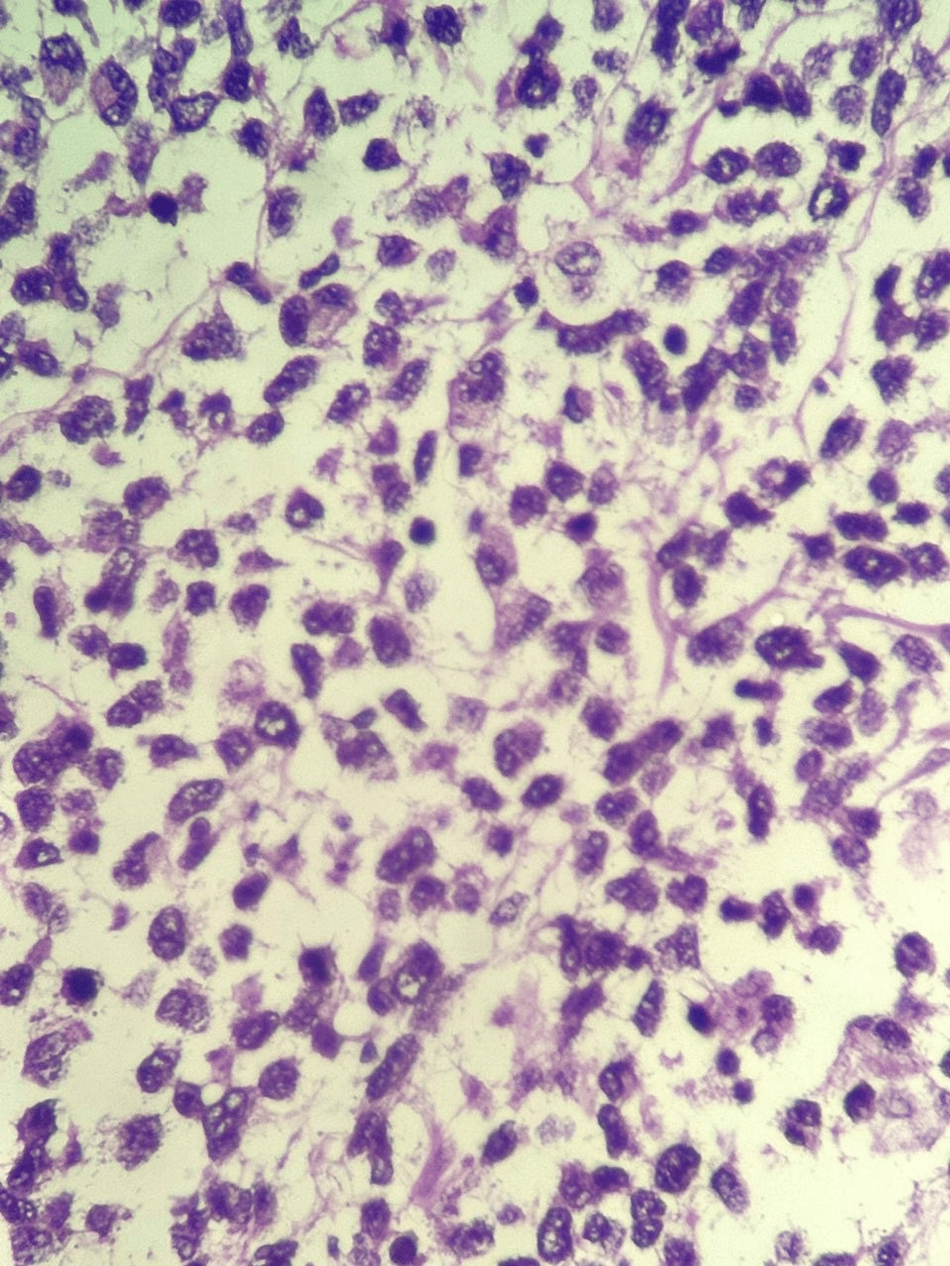
Pathological examination. Diffuse infiltration of oval, uneven cells, eosinophilic cytoplasm, and hyperchromatic, slightly eccentric nuclei.

**Figure 2:**
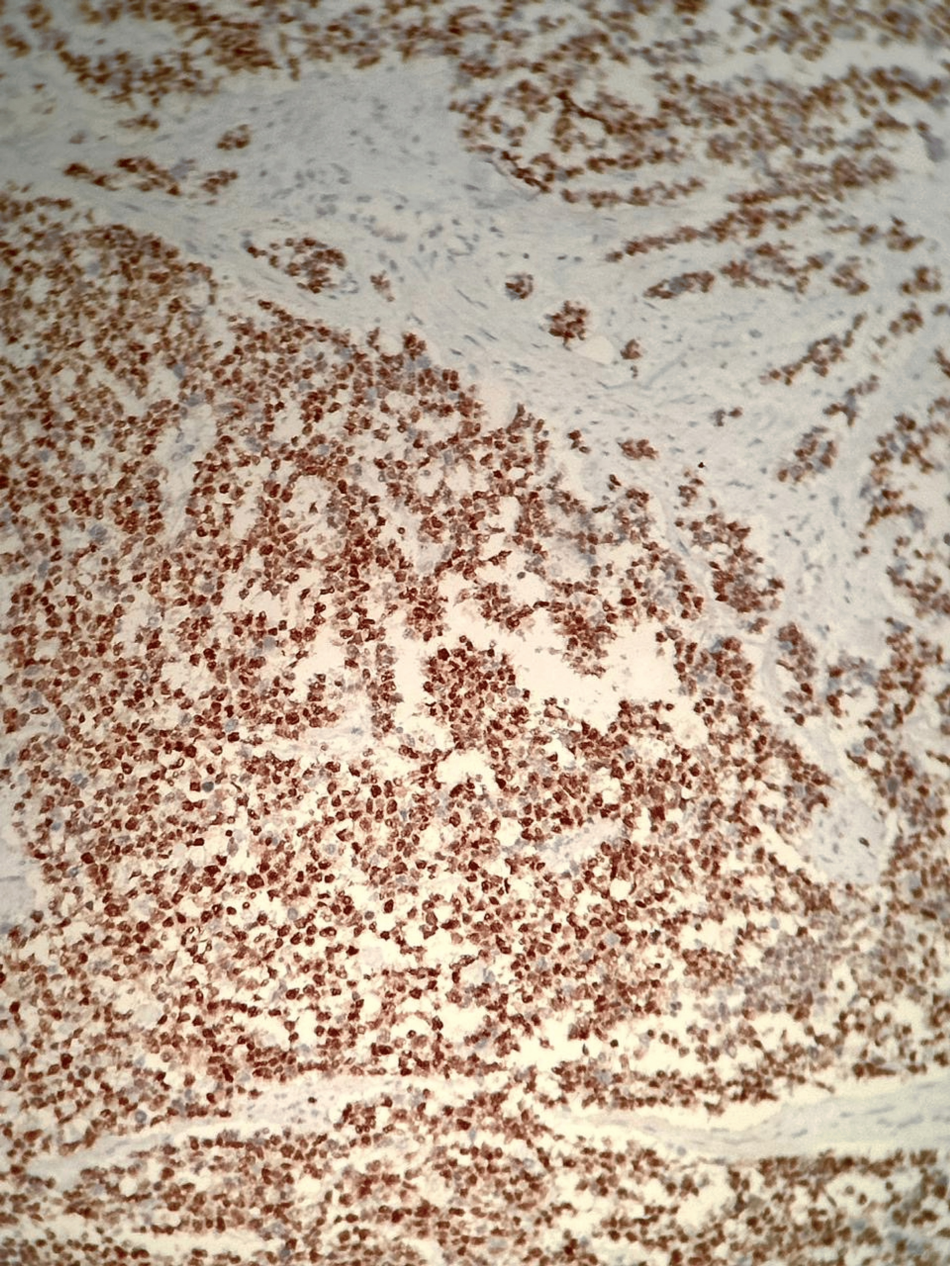
Immunohistochemical examination. Testicular slide demonstrating positivity for myogenin.

**Figure 3:**
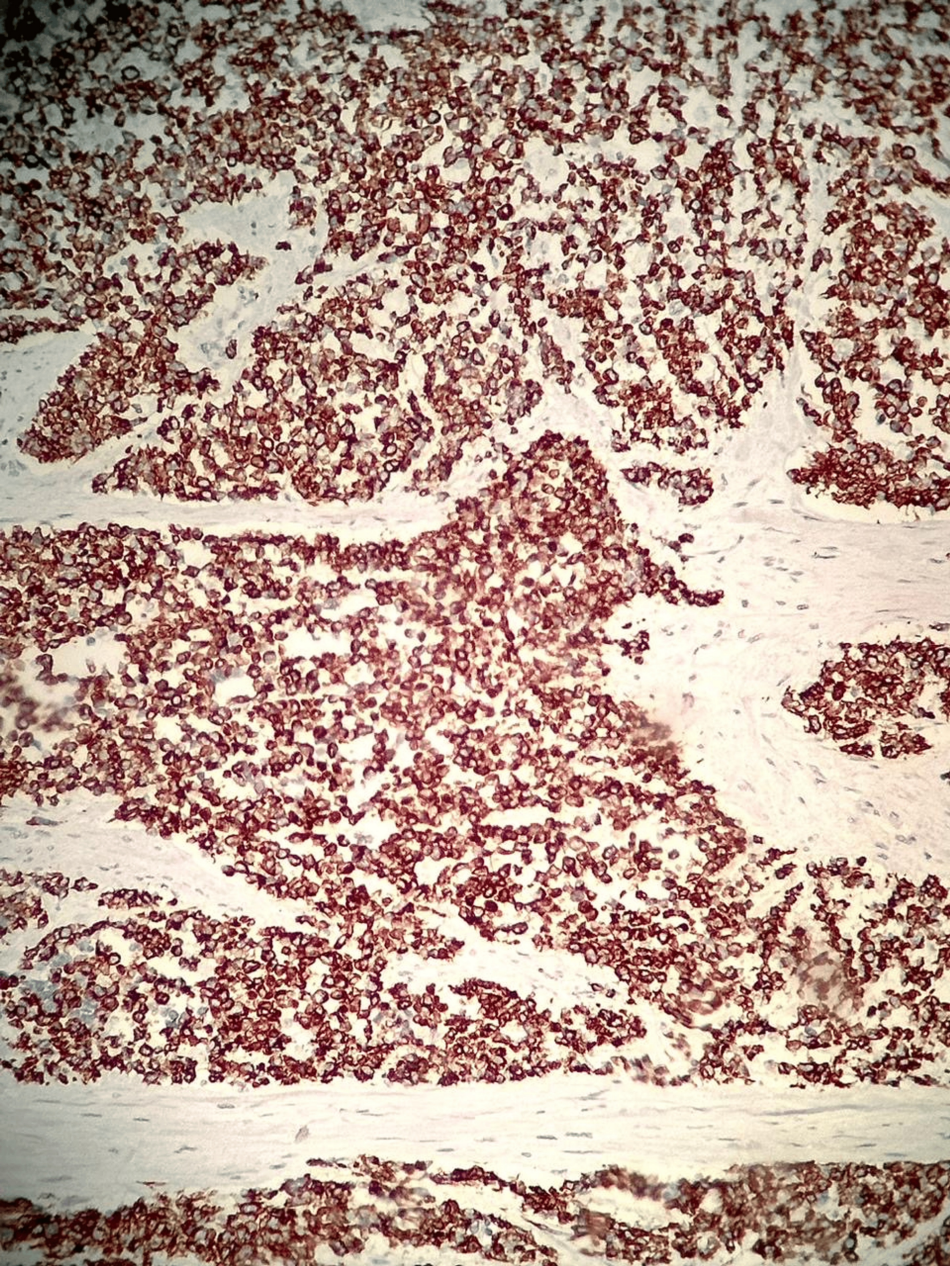
Immunohistochemical examination. Testicular slide demonstrating positivity for desmin.

A 37-year-old male patient sought a urologist after noticing the growth of the left testicle over the past couple of years. He has had an enlargement of the scrotum for approximately two years. After undergoing an orchiectomy in March 2023, he was referred to a clinical oncologist. Pathological evaluation of the left testicle described a classical seminoma measuring 6.5 cm, with invasion of the rete testis and vascular invasion (pT2). Laboratory tests showed no alterations in typical tumor markers for germ cell tumors. Imaging studies conducted in April 2023 revealed lymphadenopathy in the left superior paratracheal chain, para-aortic, subaortic, and left para-aortic areas. Following a review of the slides (Figure 1) and immunohistochemical examination, which showed diffuse positivity for myogenin (Figure 2), desmin (Figure 3), and CD 56, the diagnosis was high-grade sarcoma with features supporting the diagnosis of rhabdomyosarcoma. To confirm the diagnosis, molecular testing was performed, which revealed PAX3-FOXO1 fusion. A PET-CT was requested, which indicated lymphadenopathy with a slight metabolic increase in the retroperitoneum and the para-aortic and inter-aortocaval chains, suggesting secondary neoplastic involvement.

## Discussion

Rhabdomyosarcomas are aggressive neoplasms that typically occur in children. Paratesticular involvement is rare in adults, representing only 3.3% of soft tissue sarcomas in individuals over 19 years old, and these account for just 1% of all adult malignancies [4]. This type of neoplasm can present as an intra-scrotal mass or with scrotal enlargement, which may be either painless or painful [3,5]. In our patient, the lesion was painless, with an evolution of approximately two years, which delayed diagnosis and allowed for metastatic dissemination. In some cases, there may be compression of the testicle or epididymis, leading to confusion with differential diagnoses such as hydrocele or testicular neoplasms [1,5,6]. Diagnosis can be made through imaging studies; the initial assessment is often conducted with scrotal color Doppler ultrasound, although it may not be conclusive. Therefore, computed tomography or magnetic resonance imaging can be used to evaluate the site, dimensions, and distant metastases of the primary lesion [6]. PET-CT can also be used to gather accurate information about distant metastases [2]. The data provided through imaging becomes even more valuable as there are no specific serum markers for this tumor. Levels of human chorionic gonadotropin and alpha-fetoprotein can be found normal [5,6,7]. In our patient, the initial diagnosis was testicular seminoma, which, after reevaluation of the surgical specimen and inclusion of greater sampling, allowed for localization of the tumor as paratesticular and raised differential diagnoses such as dedifferentiated liposarcoma, neuroendocrine tumors, melanoma, and rhabdomyosarcoma. Immunohistochemistry showed positivity for myogenin, desmin, and CD56, which allowed for the diagnosis of rhabdomyosarcoma. Confirmation of the rhabdomyosarcoma subtype was made by the fusion of FOXO1, which is observed in up to 85% of cases. Patients with the PAX3-FOXO1 fusion have a significantly worse prognosis compared to patients without this fusion [8].

During the 19th century, Guersant described a polypoid tumor arising in the vagina of a young woman (because it resembled a bunch of grapes, it was given the name botryoid sarcoma). Horn and Enterline attempted, in 1958, to classify rhabdomyosarcomas for the first time, when they collected 39 cases and classified them into four distinct morphological variants: botryoid, embryonal, alveolar, and pleomorphic [2,9]. In 1995, the Intergroup Study of Rhabdomyosarcoma (IRS) proposed a new classification of rhabdomyosarcomas, taking into account the prognosis. The division was made as follows: I) Good prognosis (botryoid and spindle cell rhabdomyosarcoma); II) intermediate prognosis (embryonal rhabdomyosarcoma); III) poor prognosis (alveolar rhabdomyosarcoma and undifferentiated sarcoma); and IV) unevaluable prognosis (rhabdomyosarcoma with rhabdoid features) [5]. Furthermore, the rise of immunohistochemistry in the 1990s culminated in the use of specific markers for skeletal muscle phenotype, including desmin, myogenin, and myod1 [9]. This led to a more accurate and reliable diagnosis of rhabdomyosarcoma. Subsequently, the identification of PAX3-FOXO1 and PAX7-FOXO1 gene fusions refined the classification, as it made it feasible to place fusion-positive tumors in the alveolar subtype, regardless of cytomorphology. Furthermore, such refinement has prognostic relevance and contributes to gene expression profiles [8].

In the EIR classification for this disease, as in other types of cancer, it is necessary to pay attention to the presence of metastases since this is a fundamental milestone. In our patient, the stage was IV, without having received any previous treatment, and he evolved with a reserved prognosis, resulting in death, despite receiving treatment with chemotherapy. First, it was managed with VAC (vincristine + actinomycin D + cyclophosphamide), according to the RMS 2005 protocol of the European Pediatric Soft Tissue Sarcoma Study Group (version 1.3). However, after progression to lymph nodes and bones (between July 2023 and December 2023), he began receiving a protocol with Ifofosfamide + Etoposide + Carboplatin (between January 2024 and May 2024). After this period, he began palliation, dying in June 2024. Eighteen months elapsed between diagnosis and death, demonstrating the poor prognosis of the disease. Patients classified as group IV have a poor prognosis, with a five-year survival rate of 22.2% (compared to 94.6% in groups I to III). Given the scarcity of descriptions in the literature related to rhabdomyosarcomas, there are no protocols for specific management in adults, and it is necessary to resort to therapeutic guidelines from pediatric protocols in these situations [1].

## Conclusions

Although rhabdomyosarcomas are not common in adults, they certainly deserve attention since their presentation is highly variable and complex and may involve molecular conditions that are still poorly studied, which directly interfere with the prognosis. Therefore, we reinforce the need to elucidate these mechanisms with further research in order to provide better support to patients affected by this disease.
